# BaSAR—A tool in R for frequency detection

**DOI:** 10.1016/j.biosystems.2012.07.004

**Published:** 2012-10

**Authors:** Emma Granqvist, Matthew Hartley, Richard J. Morris

**Affiliations:** a Department of Computational & Systems Biology, The John Innes Centre, Norwich Research Park, Norwich NR4 7UH, UK; b Department of Cell & Developmental Biology, The John Innes Centre, Norwich Research Park, Norwich NR4 7UH, UK

**Keywords:** Frequency, Software, Bayesian, R

## Abstract

Many biological processes are periodic, for example cell cycle expression, circadian rhythms and calcium oscillations. However, measured time series from these processes are commonly short and noisy, and finding frequencies in such data can be challenging. Here we present BaSAR, Bayesian Spectrum Analysis in R, a package for extracting frequency information from time series data. The software uses advanced techniques of Bayesian inference that are well suited for handling typical biological data. The core functions are designed for detecting a single key frequency, without the need for data pre-processing such as detrending. The package is freely available at CRAN – The Comprehensive R Archive Network: http://cran.r-project.org/web/packages/BaSAR.

## 1 Introduction

Periodic phenomena are common in biology, over scales that range from fractions of a second to many years and from the molecular to the population level (Murray, 2003; Goldbeter, 1997). In cell biology, self-sustaining oscillations arise in many processes, including cytoskeleton dynamics, cell cycle gene expression, bacterial movements, auditory hair bundles, spatial protein distribution such as Min in *Escherichia coli*, and gene expression following circadian clock rhythms (Kruse and Jülicher, 2005).

Much work in theoretical systems biology has been devoted to the derivation and study of equations that give rise to sustained periodicity. Similarly, on the data analysis side, the development of sophisticated pattern recognition techniques for frequency detection has received much attention. A common technique, the Fourier transform, is based on the representation of any integrable function by a sum of sines and cosines. These periodic basis functions can be employed to probe the strength of frequencies in a given time series. However, the underlying assumptions for optimality of this approach include uniformly sampled, long, stationary, harmonic signals that have either no or white noise. These conditions are rarely met in biology. Therefore, techniques for detrending and noise reduction are common, but these convolute the signal, causing information loss (MacKay, 2003; Jaynes and Bretthorst, 2003). An alternative approach is to take all known effects into consideration but to integrate over the unknowns in the system. Bayesian techniques provide the appropriate framework for carrying out such marginalizations over joint probability distributions.

The advantages of using the Bayesian approach in data analysis has been documented in a number of cases (see e.g. Kotyk et al., 1992; Baldi and Long, 2001; Sivia and Skilling, 2006; Huelsenbeck and Ronquist, 2001). Here we describe our implementation of Bayesian Spectrum Analysis (Bretthorst, 1988) that offers automated background model selection and local, high-resolution frequency detection, without the need for pre-processing the data.

### 1.1 Approach

By placing the problem of frequency detection in the framework of Bayesian inference, the known and well-documented problems of Fourier analysis (see e.g. Gibbs, 1899; Bracewell, 1978; Jaynes and Bretthorst, 2003) can be overcome. This idea was pioneered by Bretthorst (1988) and was applied with success to nuclear magnetic resonance data. We recently developed this approach further by combining it with nested sampling to calculate the evidences for model comparison (Sivia and Skilling, 2006). We also introduced the generation of local frequency information (Granqvist et al., 2011). We employed this approach for the analysis of circadian clock data and calcium oscillations. The software that we present here builds on these developments and makes them easily accessible to a wider community.

We summarise the main points of the methods and refer to Bretthorst (1988) and Granqvist et al. (2011) for further details. In the following, we assume that the data, *D*, are given at *N* discrete time points, *t*_*i*_, *D* = {*d*(*t*_1_), …, *d*(*t*_*N*_)}. There is no requirement for these data to be equally spaced. The data can be modelled as a sum of the underlying signal, *s*(*t*_*i*_), a background trend, *g*(*t*_*i*_), and the noise present in the system, *e*(*t*_*i*_),

(1) $$d(t_{i})=s(t_{i})+g(t_{i})+e(t_{i}).$$

The signal can be approximated by a linear combination of *m*_*s*_ model functions, *ψ*_*i*_, parameterized by the angular frequency *ω*:

(2) $$s(t_{i})=\sum_{j=1}^{m_{s}}a_{j}\psi_{j}(\omega ,t_{i}),$$

in which $\text{a}=\{a_{1},\ldots ,a_{m_{s}}\}$ are the expansion coefficients. In the provided software, the harmonic functions sin(*ωt*) and cos(*ωt*) are used as default model functions. Similarly, any background functions that are present can be approximated by a set of trend functions that are independent of *ω*. Legendre polynomials are used for this purpose.

Following Bayes’ rule, the posterior probability distribution over the angular frequency *ω* for a given model, *H*, is given by

(3) $$P(\omega |D,H)=\frac{P(D|\omega ,H)\;P(\omega |H)}{P(D|H)},$$

where *P*(*D*|*ω*, *H*) is the likelihood, *P*(*ω*|*H*) the prior distribution over *ω*, and *P*(*D*|*H*) the evidence. Eq. (3), combined with the model presented in Eqs. (1) and (2), gives the posterior probability distribution over *ω*. After assigning priors, integrating out amplitudes and noise levels and calculating the likelihood function, the posterior has been shown (Bretthorst, 1988) to be proportional to

(4) $$P(\omega |D,H,I)\propto {\left[1-\frac{m{\bar{h}}^{2}}{N{\bar{d}}^{2}}\right]}^{(m-N)/2},$$

in which ${\bar{h}}^{2}$ is the mean-square of the data projected onto the orthonormal model functions, *ϕ*_*j*_, ${\bar{h}}^{2}=(\sum_{j=1}^{m}h_{j}^{2})/m$, where $h_{j}=\sum_{i=1}^{N}d_{i}\phi_{j}(\omega ,t_{i})$. This set of model functions can include background functions in addition to the signal model functions in Eq. (2) (Bretthorst, 1988). Periodic data and a good model will result in a high probability peak in the posterior distribution at the appropriate frequency.

To compare different models, we use posterior model ratios (Bretthorst, 1988). A given model of the signal, *H*_*i*_, can be compared to an alternative model, *H*_*j*_, by calculating their model ratios,

(5) $$\frac{P(H_{i}|D,I)}{P(H_{j}|D,I)}=\frac{P(H_{i}|I)P(D|H_{i},I)}{P(H_{j}|I)P(D|H_{j},I)},$$

where *H*_*i*_ represents the model with fewer parameters. When the ratio is above one, the simpler model is preferred (MacKay, 2003).

To calculate the evidence, the normalising component in Eq. (3), we use the method of nested sampling. This is a Bayesian variant of the Markov Chain Monte Carlo (MCMC) algorithm that concentrates its sampling efforts in high likelihood regions of parameter space (Sivia and Skilling, 2006). The algorithm focuses on the computation of the evidence, whilst at the same time generating samples from the posterior distribution. By transforming the problem to likelihood space, high-dimensional integration can be reduced to a sorting task. Random samples are taken from the prior and by rejecting the point with the worst likelihood, the algorithm iteratively contracts the spread of samples around high likelihood regions of parameter space. One of the remaining samples is chosen to generate a new sample by taking MCMC steps around it in search of a higher likelihood value. Then the new samples are again sorted and the worst point rejected. This process is iterated until a given number of posterior samples have been generated.

## 2 Software description

We have implemented the above methodology in R (R Development Core Team, 2008), This package is suitable for biologists who wish to determine whether their data contain periodic features. The package can deal with data with background trends, cases where the period changes over time, or when the data have nonuniform sampling intervals. It is also well suited for cases where a high resolution of the frequency is needed. The current version only deals with frequency searches in one dimension. The key functions are listed in Table 1, and presented briefly below. A tutorial of the package can be found in the supplementary material.

### 2.1 Key functions

BaSAR.post returns a normalised posterior probability distribution over the chosen range of frequency (*ω*). This is invoked in the manner:

BaSAR.post(data, start, stop, nsamples, nbackg, tpoints) where data is the time series as a 1D vector, start-stop is the range of the period that is of interest (in seconds), nsamples is the number of samples that will be calculated from the posterior, and tpoints is the vector of time points when the data were sampled (in seconds). The interval between the time points does not need to be uniform. BaSAR.nest calculates the evidence using nested sampling. Direct comparison of evidences can be used to evaluate models.

BaSAR.modelratio is a model comparison method that uses model ratios to allow the user to compare two models with different background functions. This procedure has been automated in BaSAR.auto. For time series in which the dominant frequency changes over time, BaSAR.local can be used to calculate the local frequency by windowing.

The outputs from all functions are the posterior probability distribution over *ω*. If the user wants to see the results over period instead, there is a helper-function for this called BaSAR.plotperiod.

### 2.2 Parameters and priors

The core model parameters that the user needs to specify are the period interval of interest, the number of samples over this interval, and the number of background functions. The period interval of interest might vary widely depending on the biological system, and must be decided from case to case. The choice of number of samples is a trade-off between frequency resolution and time of computation, but 100–500 samples is often a good starting point. The number of background functions needed can be estimated by using the automated model selection functions such as BaSAR.auto. The total number of model functions, *m*_*s*_ in Eq. (2), is two for the basic sine and cosine, plus any added background functions.

Model parameters that are not of principal interest, such as amplitudes and noise level, are assigned suitable priors (Jeffreys prior for noise levels, uniform prior for amplitudes) and then integrated over following Bretthorst (1988). At the moment they are not part of the software output.

## 3 Application examples

Test cases were presented in Granqvist et al. (2011) on both real and simulated data. Here we introduce two further examples to illustrate the global and local frequency detection ability of BaSAR.

### 3.1 Cell cycle genes

**Fig. 1:**
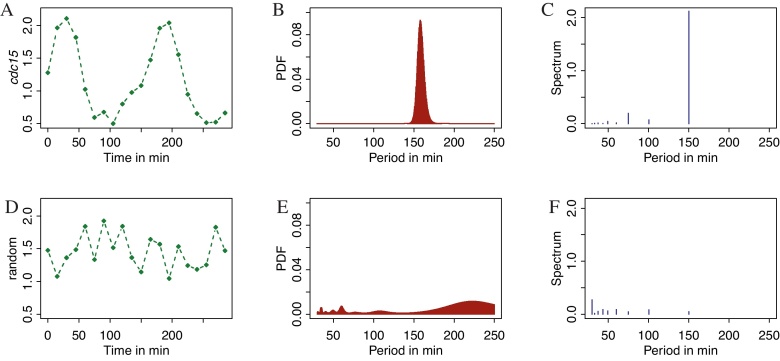
Example results from BaSAR.post. The left column (green) shows the time series, the middle (red) shows the BaSAR posterior probability density function (PDF) over the sampled range, and the right column (blue) show the time series Fourier spectra for comparison. (A) Time series of *cdc15* expression. (B) PDF of *cdc15*. (C) Fourier spectrum of *cdc15*. (D) time series with random numbers between 1 and 2. (E) PDF of the random time series. (F) Fourier spectrum of the random time series. See main text for details on the data. (For interpretation of the references to colour in this figure legend, the reader is referred to the web version of the article.)

We demonstrate BaSAR on gene expression data from cell cycle phases in fission yeast, *Schizosaccharomyces pombe*, taken from Rustici et al. (2004). These time series are short, and have long intervals between data points. Many genes peak in expression during specific phases of the cell cycle, resulting in cyclic expression profiles (Breeden, 2003). Fig. 1A shows one such gene's expression profile, *cdc15*. This gene is known to be expressed periodically and peaks in M phase, with a characterized role in the actin ring formation of cell division (Fankhauser et al., 1995; Zilahi et al., 2000). A clear peak can be seen in the posterior distribution output from BaSAR.post (Fig. 1B). It should be noted that only one time series for the gene is included here (from Elutriation 1), not the complete data set that the original study used. A Fourier period spectrum is shown for comparison (Fig. 1C). Whilst the Fourier spectrum performs well, the accuracy is limited by the sampling that is a function of the given time points, leading to a slight shift in frequency peak in this example. Furthermore, Fourier spectra requires uniformly sampled data points. A simulated time series consisting of uniform random numbers between 1 and 2 is shown, along with its BaSAR results and its Fourier spectrum (Fig. 1D–F), to demonstrate a posterior distribution with no evidence for periodicity.

### 3.2 Calcium oscillations

**Fig. 2:**
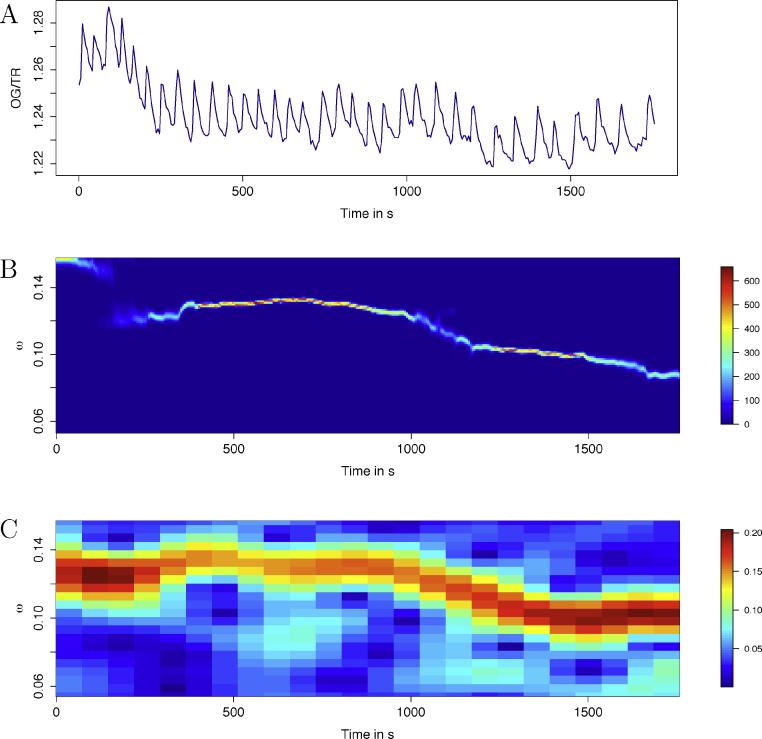
Example results from BaSAR.local. (A) Calcium oscillations measured in *M. truncatula* root hair cells. (B) The 2-dimensional PDF over time and angular frequency (*ω*), showing that oscillation frequency varies over time. (C) The 2-dimensional Fourier spectra from a windowed Fourier transform, for comparison.

In the symbiosis between legumes and nitrogen-fixing bacteria (rhizobia), calcium oscillations are induced in the plant root cells during the initial signalling stages. These calcium signals occur in and around the nucleus, and are essential for the symbiosis to establish (Oldroyd and Downie, 2006). The oscillations often start with a rapid period (of approximately 1 min) and often slow down over time as the signal continues. The data also contain background trends due to fluorescence bleaching. An example time series is shown in Fig. 2A. In Fig. 2B, the result of analysing this time series with BaSAR.local is shown, demonstrating that this package copes with the background trend as well as shows the signal period changes over time, without need for pre-processing such as detrending. Good results can be achieved by a windowed Fourier spectra (Fig. 2C) but only after detrending, which was done using a moving average (Brockwell and Davis, 2002). BaSAR delivers superior results (Fig. 2B) and without the need for data pre-processing.

## Figures and Tables

**Table 1:** Key functions in the BaSAR package.

Function	Description
BaSAR.post	Normalized posterior probability distribution
BaSAR.nest	Posterior and evidence using nested sampling
BaSAR.modelratio	Model comparison for background trends
BaSAR.auto	Automated BaSAR.modelratio
BaSAR.local	2D posterior over time and *ω* by windowing
