# Supplementary material for: BaSAR—A tool in R for frequency detection
Source: Biosystems. 2012 Oct;110(1):60–3. doi: 10.1016/j.biosystems.2012.07.004 (PMC3462997; doi:10.1016/j.biosystems.2012.07.004)
Supplement: Supplementary file 1 [file mmc1.pdf]

Tutorial for BaSAR - a tool in R for frequency detection

Installing BaSAR

The package and a manual are available at http://cran.r-project.org/web/packages/BaSAR/. To install from the R command line, type the following:

```
install.packages("BaSAR")
library(BaSAR)
```

The package should now be installed and ready to use. An additional library is useful for plotting. To load this type:

```
library(fields)
```

Basic functions

The data sets that we will use have been loaded along with the package, and the first is called `tutorial`. The first column in this data set is the time vector, and the rest are simulated time series. The time points are evenly sampled, but this is not a requirement of the package.

BaSAR.post

The basic function in BaSAR is `BaSAR.post`, which is used to compute the posterior distribution over the frequency. It is used in this manner:

```
BaSAR.post(data, start, stop, nsamples, nbackg, tpoints).
```

The function expects four parameters that define the interval in seconds over which the posterior probability for the period is computed (`start, stop`), the number of samples to use in the interval (`nsamples`), the number of background functions (`nbackg`) as well the data points (`data`) and of time points for the data (`tpoints`).

The first dataset consists of the sine wave of angular frequency 0.5 rad/s with added random noise. The plot of the time series is shown in Figure 1, left panel. To plot this data, type:

```
plot(tutorial[,2], type="l", col="blue", xlab="t", ylab="d(t)")
```

We need to specify the period range we wish to explore. In this example, we have chosen the generous range of 6s to 600s. The number of samples was set to 100. A higher number increases the computational time, and a lower number decreases the accuracy of the frequency estimate. From the plot of the time series no background trend is detectable, so there is no need to add background model functions. This parameter is therefore set to zero.

Run `BaSAR.post` with these settings. It will return the normalized posterior distribution of angular frequency over the chosen interval.

```
r <- BaSAR.post(tutorial[,2], 6, 600, 100, 0, tutorial[,1])
```

Plot the resulting posterior density function, and this should look like the right panel of Figure 1.

```
plot(r$omega, r$normp, xlim=c(0:1), type="h", col="red", ylab="PDF",
xlab=expression(omega))
```

The high peak is at the correct angular frequency of $\omega = 0.5$.

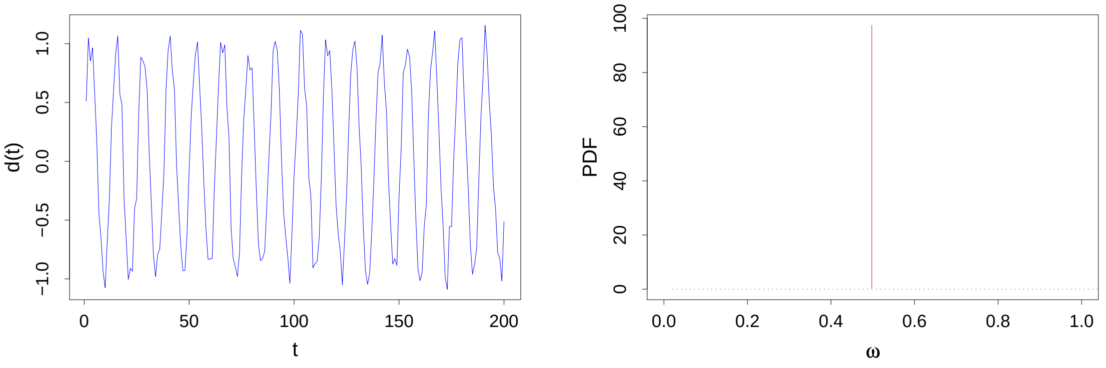

Figure 1: BaSAR.post

BaSAR.fine

The function `BaSAR.fine` is useful when a higher resolution of the frequency is desired. It identifies the highest probability peak, and then samples again in a narrower interval around this peak. It is used as `BaSAR.post`:

```
BaSAR.fine(data, start, stop, nsamples, nbackg, tpoints)
```

The second time series that we will analyze is is also a sine wave with an angular frequency of 0.5, but with a much higher noise level. To go through the example, type the following on the R command line:

```
plot(tutorial[,3], type="l", col="blue", xlab="t", ylab="d(t)")
r <- BaSAR.fine(tutorial[,3], 6, 600, 100, 0, tutorial[,1])
plot(r$omega, r$normp, xlim=c(0:1), type="h", col="red", ylab="PDF",
xlab=expression(omega))
```

The result can be seen in Figure 2. Despite the higher noise level, BaSAR identifies the correct angular frequency.

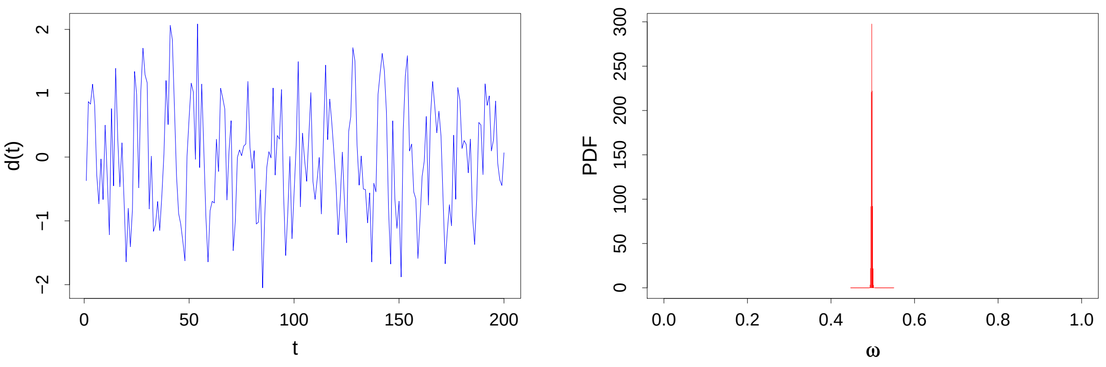

Figure 2: BaSAR.fine

BaSAR.auto

Many types of data, especially in biology, contain background trends or fluctuations that are irrelevant to the signal of interest. In BaSAR, it is possible to account for such background trends by adding background model functions to the model. In this way, we avoid involving detrending techniques in our analysis. The function `BaSAR.auto` is an automated way of choosing how may background functions are optimal for the time series at hand, by using Bayesian model ratios. The user only needs to specify an upper limit, `nbackg`. In all else, the function is very similar to the basic `BaSAR.post`:

```
BaSAR.auto(data, start, stop, nsamples, nbackg, tpoints)
```

Plot the time series, the fourth column of `tutorial`. This is again a sine wave with angular frequency of 0.5 and a small amount of random noise, but there is also a background trend added.

```
plot(tutorial[,4], type="l", col="blue", xlab="t", ylab="d(t)")
```

The plot is shown in the left panel of Figure 3. It is a strong background trend, but not very complicated, so a few background functions should suffice. We choose four as the upper limit, and for the other parameters we keep the same settings that we have used before.

```
r = BaSAR.auto(tutorial[,4], 6, 600, 100, 4, tutorial[,1])
plot(r$omega, r$normp, xlim=c(0:1), type="h", col="red", ylab="PDF",
```

```
xlab=expression(omega))
```

The result shows that BaSAR has managed to find the correct angular frequency, despite the strong background present. If you type `r$model` you will see how many background model functions were chosen by the software in this instance (two). Here you will also be warned if your upper limit was reached, and in that case you should increase it.

Model functions can also be added manually and their impact evaluated using the `BaSAR.modelratio` function. For more information on this function, see the package manual on the CRAN website.

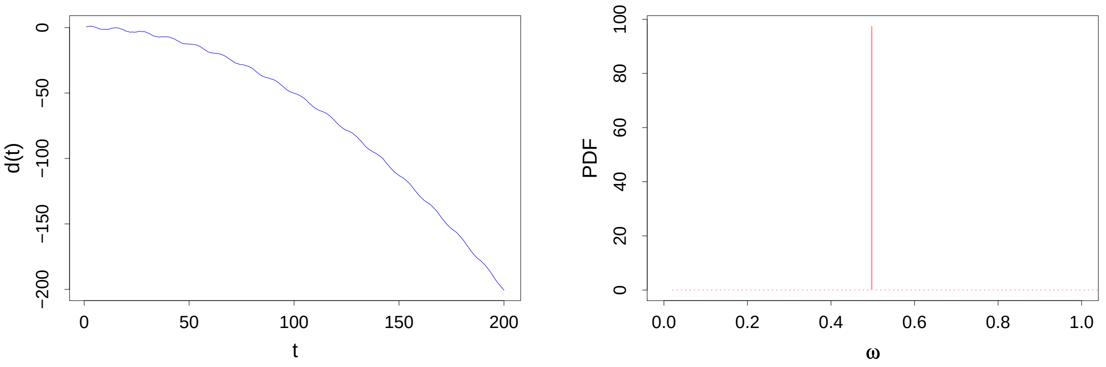

Figure 3: BaSAR.auto

BaSAR.local

Some frequencies change over time. In this case, there is a windowed version of `BaSAR.post` called `BaSAR.local` that is convenient." to "To handle frequencies that vary over time, `BaSAR.local` is provided which uses as windowed version of `BaSAR.post`. Here an additional parameter needs to be specified, namely the size of the window. This may require some trial and error from the user since the optimal window size will vary with the data. The function is used in this manner:

```
BaSAR.local(data, start, stop, nsamples, tpoints, nbackg, window)
```

As before, start by visually inspecting the time series, then choose the settings and run `BaSAR.local`. The time series is now the fifth column of `tutorial`, and is a sine wave that starts with an angular frequency of 0.5 but this increases with every time point.

```
plot(tutorial[,5], type="l", col="blue", xlab="t", ylab="d(t)")
r <- BaSAR.local(tutorial[,5], 2, 30, 100, tutorial[,1], 0, 10)
image.plot(tutorial[,1],r$omega,r$p,ylab=expression(omega),xlab="t",
col=rev(heat.colors(100)))
```

The result here is a two-dimensional posterior distribution over the angular frequency and time, and is shown in the right panel of Figure 4.

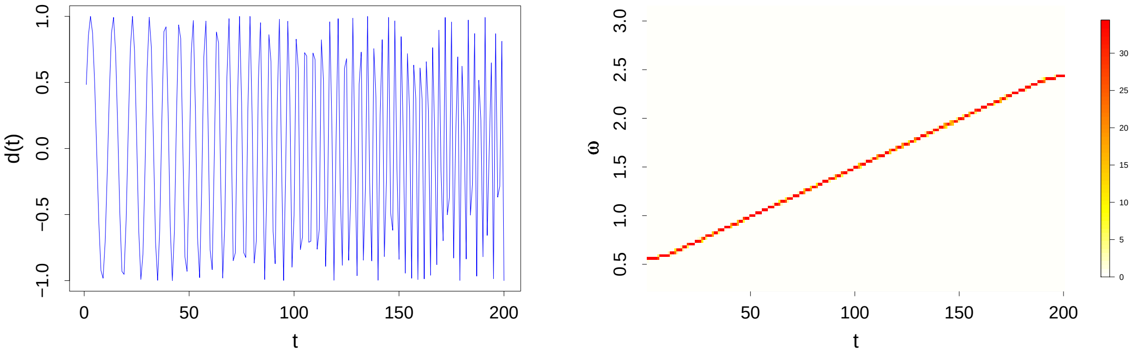

Figure 4: BaSAR.local

An example with real data

The last example is a time series showing oscillations in calcium concentrations, and there are two points that need attention. The data contain a background trend, which is separate from the oscillations that we are interested in. Also, the frequency seems to slow down over time. Therefore, we will use `BaSAR.local`, and add background model functions to account for the background trend.

Start by plotting the time series, as shown in Figure 5. The first and second columns in the second data set `tutorial.2` represent the time and data vectors respectively:

```
plot(tutorial.2[,1],tutorial.2[,2],type="l",col="blue",xlab="t",ylab="d(t)")
```

One background function will be sufficient, since the trend is not very strong. For the other parameters, a period between 40 and 120 seconds looks likely from a visual inspection, and we choose a window size of 50 data points. Now we will run BaSAR.local and plot the result.

```
r <- BaSAR.local(tutorial.2[,2], 40, 120, 100, tutorial.2[,1], 1, 50)
image.plot(tutorial.2[,1],r$omega,r$p,ylab=expression(omega),xlab="t",
col=rev(heat.colors(100)))
```

The result in the right panel of Figure 5 shows the decreasing period and also demonstrates the higher uncertainty that comes with real biological data, compared to the simulated case.

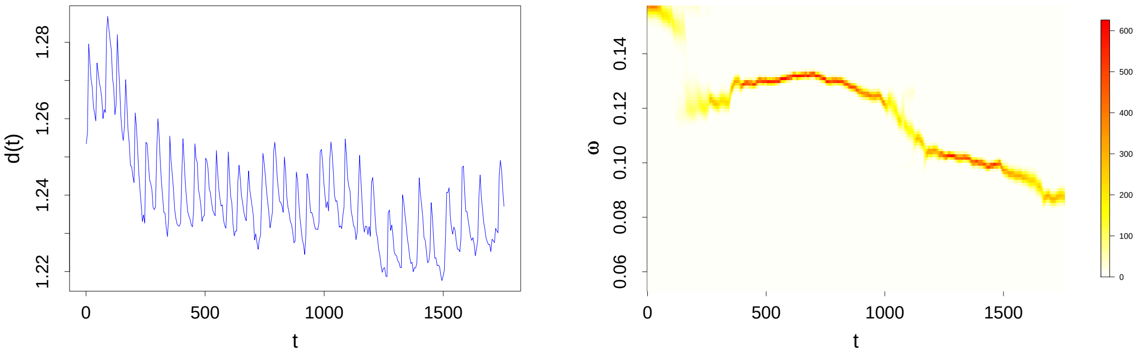

Figure 5: BaSAR.local on calcium oscillation data
